# Prevalence of use and interest in using glucagon-like peptide-1 receptor agonists for weight loss: a population study in Great Britain

**DOI:** 10.1186/s12916-025-04528-7

**Published:** 2026-01-08

**Authors:** Sarah E. Jackson, Jamie Brown, Clare Llewellyn, Oliver Mytton, Lion Shahab

**Affiliations:** 1 Department of Behavioural Science and Health, University College London, 1-19 Torrington Place, London, WC1E 7HB UK; 2 Institute for Child Health, University College London, London, UK

**Keywords:** Semaglutide, Liraglutide, Tirzepatide, Ozempic, Wegovy, Saxenda, Mounjaro

## Abstract

**Background:**

This study aimed to assess the prevalence of glucagon-like peptide-1 (GLP-1) receptor agonist use and interest in using medications for weight loss among adults (≥ 18 years) in Great Britain.

**Methods:**

Nationally representative household survey, January–March 2025 (*n* = 5893). Participants were asked whether they had used medication in the past year to manage type 2 diabetes (excluding insulin), reduce the risk of heart disease, or support weight loss and, if so, whether they had used any of five specific GLP-1 or dual GLP-1/glucose-dependent insulinotropic polypeptide (GIP) receptor agonists. Those who had not used medication to support weight loss in the past year were asked how likely they would be to consider doing so in the next year. Estimates were reported stratified by participant characteristics and extrapolated to the national population.

**Results:**

Overall, 2.9% [2.4–3.4%]— approximately 1.6 million adults—reported using a GLP-1 or GLP-1/GIP medication to support weight loss in the past year, with 1.7% [1.4–2.1%] (~ 910,000 adults) using them exclusively for this purpose. Of those who used them exclusively for weight loss, the majority (91.4% [85.6–97.2%]) reported using medications licensed for this purpose in Great Britain, most commonly Mounjaro (tirzepatide; 80.2% [71.9–88.6%]). Of those who had not used weight-loss medication in the past year, 6.5% [5.7–7.3%] (~ 3.3 million adults) expressed an interest in doing so in the next year. Use and interest were more prevalent among women, people in mid-life, and those reporting past-month psychological distress. Interest was also higher among people facing greater socioeconomic disadvantage.

**Conclusions:**

In the first quarter of 2025, an estimated 4.9 million adults in Great Britain—nearly one in ten—either had recently used a GLP-1 or GLP-1/GIP medication to support weight loss or were interested in doing so in the near future. A substantial minority reported using a type of GLP-1 medication that was not licensed for weight management, suggesting off-label use. Interest was particularly high among less advantaged socioeconomic groups, while use was similar across groups, highlighting the importance of addressing equity in access. These findings underscore the need to monitor who is accessing these medications and to ensure their safe, appropriate, and equitable provision.

**Supplementary Information:**

The online version contains supplementary material available at 10.1186/s12916-025-04528-7.

## Background

The high prevalence of obesity in Great Britain presents significant public health challenges, increasing the risk of chronic diseases and placing a substantial burden on the National Health Service (NHS) [1, 2]. Advancements in pharmacotherapy—particularly glucagon-like peptide-1 (GLP-1) receptor agonists and dual GLP-1/glucose-dependent insulinotropic polypeptide (GIP) agents (henceforth referred to as GLP-1 and GLP-1/GIP medications)—have emerged as a promising tool for weight management. Originally developed and increasingly used for the treatment of type 2 diabetes, these medications have proven highly effective in promoting weight loss, at least during treatment [3–5], sparking widespread public interest. They also have cardio-protective effects, reducing the risks of heart attack, stroke, and cardiovascular mortality [6, 7] and may help to reduce substance use disorders (e.g. alcohol and tobacco use) [8], but safety of long-term use is currently uncertain [9]. Relatively little is known about the current prevalence of the use of GLP-1 and GLP-1/GIP medications in Great Britain [10], potential future demand, and how interest and usage patterns vary across different population subgroups. Understanding these factors is important for informing healthcare planning and resource allocation, promoting patient safety, and addressing health inequalities [11].

In the UK, several GLP-1 medications are available, including semaglutide, liraglutide, dulaglutide, exenatide, and lixisenatide. These are supplied under various brand names, some of which have more than one indication (see [12] for a summary). In addition, tirzepatide is a GLP-1 and GIP compound. GLP-1 and GLP-1/GIP products currently licensed for weight loss in the UK—Saxenda (liraglutide), Wegovy (semaglutide), and Mounjaro (tirzepatide)—are licensed by the Medicines and Healthcare products Regulatory Agency for patients who (i) have obesity (body mass index [BMI] ≥ 30 kg/m^2^) or (ii) have a BMI in the overweight range (BMI ≥ 27 kg/m^2^) plus weight-related comorbidities, such as cardiovascular disease [12]. Clinical guidelines from the National Institute for Health and Care Excellence (NICE) [13], making recommendations to the NHS to ensure value for money, set a higher threshold for use. NICE recommend tirzepatide and semaglutide for patients with at least one weight-related comorbidity and a BMI ≥ 35 kg/m^2^ (or BMI ≥ 30 kg/m^2^ and who meet the criteria for referral to specialist weight management services, for semaglutide only) and liraglutide for patients with a BMI ≥ 35 kg/m^2^, non-diabetic hyperglycaemia, and a high risk of cardiovascular disease. In England, this would equate to 3.4 million being eligible for the medications on the NHS [14]. Given concerns about cost and resources pressure, the NHS in England plans a phased roll out, offering the drugs to 220,000 people between 2025 and 2028 [14].

Public interest in, and use of, GLP-1 and GLP-1/GIP medications for weight loss is growing rapidly. In the USA, a poll conducted in May 2024 (*n* = 1479) found that 6% of adults were currently using a GLP-1 or GLP-1/GIP medication and 12% had ever used one [15]. Among those who reported ever taking the drugs, most (61%) said they took them to manage a chronic condition such as diabetes or heart disease, but around four in ten (38%) said they took them solely to lose weight [15]. Similarly, demand for these medications in the UK appears to be high, with concern that the NHS is struggling to manage demand for weight-loss drugs [16]. Prescribing data show sharp increases in prescription of semaglutide and tirzepatide across NHS GP practices in England in 2024–2025 [17, 18]. A December 2024 poll (*n* = 2161) found that 22% of UK adults would use a GLP-1 weight-loss drug if it were available on prescription through the NHS [19], although many of these may not be eligible according to NICE guidelines. Of those surveyed, 5% had personally taken a GLP-1 medication, while 9% knew friends or family members who had used one [19].

Despite the growing interest in these medications, there is also concern about their misuse. Anecdotal evidence and adverse drug reaction reports suggest some people are using GLP-1 and GLP-1/GIP medications outside of licensed indications [12], potentially posing health risks. Additionally, gastrointestinal side effects (e.g. nausea, vomiting, diarrhoea, and constipation) are common [3, 5], serious adverse effects (e.g. pancreatitis) have been reported [11], and some people who use GLP-1 medications experience malnutrition [20–23], further raising safety considerations. As these medications become more widely used, it is essential to monitor their usage trends and to understand who is using them. With some people accessing these medications outside of the NHS, traditional systems for understanding usage (e.g. NHS prescribing data, Clinical Practice Research Datalink) cannot be relied upon to provide accurate estimates. Triangulation with nationally representative surveys is important for providing a complete picture.

Gender differences may play a role in the demand for and usage of GLP-1 and GLP-1/GIP medications. Severe obesity (BMI ≥ 40) is more prevalent among women than men (4% vs. 2% among adults in England in 2022) [2] and women are more likely to seek and receive medical treatment for weight loss [24], so they may be prescribed these drugs at higher rates than men. Additionally, societal pressures related to body image and weight management disproportionately affect women [25], which could further drive interest in GLP-1 and GLP-1/GIP medications within this group.

Equity of access to these medications is another important factor [26]. In the UK poll, just 8% said they would use these medications for weight loss if they had to pay for them privately, compared with the 22% who would take them if provided on prescription by the NHS [19]. Given it currently costs in the region of £200 per month to obtain these drugs privately [19], those with the financial means may have greater access to treatment, potentially exacerbating health inequalities. This is particularly concerning as obesity is more prevalent among socioeconomically disadvantaged groups, particularly among women, according to area level deprivation and household income [27, 28].

Given the rising public interest, potential supply challenges, and safety concerns, this study aimed to assess the current prevalence of GLP-1 and GLP-1/GIP medication use and level of interest in using these medications for weight loss among the adult population in Great Britain. We also explored differences between key population subgroups. A comprehensive understanding of these factors can help to inform healthcare policy, ensure equitable access to treatment, and safeguard patient wellbeing.

## Methods

### Pre-registration

The study protocol and analysis plan were pre-registered on Open Science Framework (https://osf.io/r2whq/).

### Design

Data were collected via the Smoking and Alcohol Toolkit Study, a monthly cross-sectional survey of a representative sample of adults (≥ 16 years) in Great Britain (i.e. England, Scotland, and Wales). The methods have been described in detail elsewhere [29–31]. Briefly, the study uses a hybrid of random probability and simple quota sampling to select a new sample of approximately 2450 adults each month. Data are collected through telephone interviews. Sample weights are calculated using raking to match the population in Great Britain. This profile is determined each month by combining data from the UK Census, the Office for National Statistics mid-year estimates, and the annual National Readership Survey [29]. Comparisons with other national surveys and sales data indicate the survey achieves nationally representative estimates of key sociodemographic variables [29].

Between January and March 2025, all participants aged ≥ 18 years in England and ~ 50% in Wales and Scotland were asked additional questions on past-year use of GLP-1 and GLP-1/GIP medications and interest in using them for weight loss. This study analysed these data.

### Measures

Past-year use of GLP-1 receptor agonists and GLP-1/GIP compounds (i.e. tirzepatide) was assessed with two questions. The first asked: ‘In the last 12 months have you taken any medication to help you with any of the following, or not?’ Participants were asked to indicate all that applied from the following response options: (a) medication for type 2 diabetes, excluding insulin, to help manage your blood sugar levels; (b) medication to lower the risk of heart disease; (c) medication to support weight loss/reduce food cravings; (d) I have not taken medication for any of these reasons. They could also respond that they could not remember. Those who reported using medication (i.e. responded a, b, or c) were then asked: ‘In the last 12 months, which, if any, of the following types of medication have you taken [for type 2 diabetes/to lower the risk of heart disease/for weight loss]?’ (a) Saxenda, containing liraglutide; (b) Ozempic, containing semaglutide; (c) Wegovy, containing semaglutide; (d) Mounjaro, containing tirzepatide; (e) Rybelsus, containing semaglutide; (f) another type of medication. Participants were asked to select all that applied. They could also respond that they did not know. Those who responded to one of the named drugs, i.e. responses (a) to (e), to the second question were considered to have used a GLP-1 or GLP-1/GIP medication.

Interest in using medication for weight loss was assessed among those who did not report using medication for weight loss in the past year (i.e. responded a, b, or d to the first question) with the question: ‘In the next 12 months, how likely, if at all, are you to consider using a weight loss medication to help you lose weight?’ Response options were as follows: (a) very likely, (b) fairly likely, (c) not very likely, (d) not at all likely, (e) I don’t need to lose weight. Participants could also respond that they did not know. We provided descriptive data for all response options. For some analyses, we dichotomised responses to a or b (likely to consider) vs. all other responses including don’t know. We note that without information on people’s BMI and underlying medical risk factors, it is not possible to determine whether this reflects a genuine medical need.

We captured a range of sociodemographic characteristics. Gender was self-reported as man, woman, or in another way; the latter group was excluded from analyses by gender due to low numbers. Age was analysed as a continuous variable. Ethnicity was categorised as white vs. minority ethnic group. Occupational social grade was categorised using National Readership Survey classifications [32] as ABC1 (includes managerial, professional, and upper supervisory occupations) and C2DE (includes manual routine, semi-routine, lower supervisory, state pension, and long-term unemployed). Financial situation was assessed with the question: ‘How well would you say you yourself are managing financially these days? Would you say you are (a) living comfortably; (b) doing alright; (c) just about getting by; (d) finding it quite difficult; or (e) finding it very difficult?’ [33]. Health-related economic inactivity was operationalised as participants reporting that they are ‘not in paid work because of long-term illness or disability’, in response to a question asking which of a list of different working statuses applies to them.

We also recorded information on participants’ health-related behaviours and mental health. Smoking status was categorised as current, former, or never smoker. Level of alcohol consumption was assessed with the Alcohol Use Disorders Identification Test—consumption (AUDIT-C; possible range = 0–12) and analysed as a continuous variable. As a general guide, AUDIT-C scores ≥ 5 indicate drinking at increasing or higher-risk levels (i.e. levels that increase someone’s risk of harm) [34]. Psychological distress was assessed using the Kessler Psychological Distress Scale (K6), which measures non-specific psychological distress in the past month (possible range = 0–24) [35, 36]. We categorised K6 scores as ‘no/low distress’ (0–4) or ‘moderate/severe distress’ (≥ 5) [35, 37]. History of eating disorders was assessed with the question: ‘Since the age of 16, which of the following, if any, has a doctor or health professional ever told you that you had…?’ Responses were dichotomised to distinguish between those who responded ‘an eating disorder’ and those who did not. Due to limited availability of funding, the question assessing eating disorders was only included in one of the three survey waves (February 2025), so analyses of this variable were restricted to those surveyed in this wave.

Full details of all measures are provided in Additional file 1.

### Statistical analysis

Data were analysed using R v.4.4.1. All analyses used weighted data. Missing data were handled using multiple imputation by chained equations. We imputed missing values under the assumption of missing at random, using the *mice* package. Five imputed datasets were generated. The imputation models included all variables used in the analysis (except history of eating disorders, given it was only assessed in one wave; the imputation process was repeated separately for this wave of data to provide complete data for analyses involving this variable). Analyses were conducted separately on each imputed dataset and results were combined across imputations using Rubin’s rules [38] to account for within- and between-imputation variance. Results were reported as pooled estimates and confidence intervals (CIs).

We estimated proportions (with 95% CIs) of adults who reported having used GLP-1 or GLP-1/GIP medication in the past year (a) for any reason, (b) to manage type 2 diabetes, (c) to reduce the risk of heart disease, (d) to support weight loss, and (e) exclusively to support weight loss (i.e. not also to manage type 2 diabetes or to reduce the risk of heart disease). We also reported these prevalence estimates stratified by the specific medication used. Among adults who reported having used a GLP-1 or GLP-1/GIP medication for weight loss in the past year (at all, and exclusively for weight loss), we estimated the proportion who used a medication licensed for weight loss in Great Britain (Saxenda, Wegovy, or Mounjaro). Among adults who had not used a medication for weight loss in the past year, we estimated the proportion who would be likely to consider using a weight-loss medication in the next year. We applied these proportions to the most recent (2023) mid-year population estimates for Great Britain [39] to approximate the number of adults using these medications in 2024 and the number who would be interested in using them in the next year.

To explore differences in (i) past-year use of GLP-1 or GLP-1/GIP medication to support weight loss and (ii) interest in using a weight-loss medication in the next year between population subgroups, we estimated these proportions stratified by gender, age, ethnicity, occupational social grade, financial situation, health-related economic inactivity, smoking status, alcohol consumption, past-month distress, and history of eating disorders. We also examined proportions within intersections of gender and each other variable (results are reported in Additional file 2). Age and level of alcohol consumption were analysed as continuous variables, modelled non-linearly using restricted cubic splines (with three knots placed at the 5, 50, and 95% percentiles) to allow for flexible associations without arbitrary categorisation. Estimates for continuous variables were predicted from unadjusted logistic regression models that tested the association of age/level of alcohol consumption with each outcome. We reported estimates for selected ages (18, 25, 35, 45, 55, 65, and 75) and AUDIT-C scores (0, 3, 5, 8, 11) as an illustrative example of differences across ages and levels of alcohol consumption. We also ran logistic regression models to analyse associations between each participant characteristic and these two outcomes, adjusted for age and gender (or just age, for gender-stratified analyses).

### Patient and public involvement (PPI)

The wider Smoking and Alcohol Toolkit Study is discussed several times a year with a diverse PPI group and the authors regularly attend and present at meetings at which patients and the public are included. Interaction and discussion at these events help shape the broad research priorities and questions. There is also a mechanism for generalised input from the wider public: each month interviewers seek feedback on the questions from all respondents, who are representative of the population in Great Britain. This feedback is limited and usually relates to understanding of questions and item options. No patients or members of the public were involved in setting the research questions or the outcome measures, nor were they involved in the design and implementation of this specific study.

## Results

A total of 5893 participants were invited to complete the GLP-1 survey module, of whom 5260 (89.3%) consented. Characteristics of the total eligible sample and those who consented are shown in Additional file 2: Table S1 alongside imputed data.

### Prevalence of GLP-1 or GLP-1/GIP medication use

Overall, 4.5% of participants reported using a GLP-1 or GLP-1/GIP medication in the past year for any reason; 2.9% reported using them to support weight loss, with 1.7% using them exclusively for weight loss (i.e. not also for type 2 diabetes or heart disease; Table 1). When extrapolated to the national population, these figures suggest that as of early 2025, approximately 1.6 million adults in Great Britain had used GLP-1 or GLP-1/GIP medications to support weight loss in the past year (53.7 million adults × 2.9%), of whom 910,000 used them exclusively for weight loss (53.7 million × 1.7%).

**Table 1:** Past-year use of GLP-1 receptor agonists and GLP-1/GIP compounds among adults (≥ 18 years) in Great Britain (*n* = 5893)

Medication used in past year	Authorised indication(s)^a^	Prevalence, % [95% CI]				
		**For any reason**	**To manage type 2 diabetes**	**To reduce the risk of heart disease**	**To support weight loss**	**Exclusively to support weight loss**
Any GLP-1 or GLP-1/GIP listed below	–	4.5 [3.8–5.1]	1.7 [1.3–2.0]	1.6 [1.3–2.0]	2.9 [2.4–3.4]	1.7 [1.4–2.1]
Saxenda, containing liraglutide	WL	0.4 [0.2–0.5]	0.2 [0.0–0.3]	0.1 [0.0–0.3]	0.1 [0.0–0.2]	0.0 [0.0–0.0]
Wegovy, containing semaglutide	WL, CRR	0.8 [0.5–1.0]	0.2 [0.1–0.3]	0.3 [0.1–0.5]	0.6 [0.3–0.8]	0.4 [0.2–0.5]
Mounjaro, containing tirzepatide	WL, T2D	2.3 [1.9–2.7]	0.6 [0.4–0.9]	0.7 [0.4–0.9]	2.0 [1.6–2.4]	1.4 [1.1–1.7]
Ozempic, containing semaglutide	T2D	1.3 [0.9–1.7]	0.6 [0.4–0.9]	0.6 [0.3–0.9]	0.5 [0.3–0.7]	0.2 [0.1–0.3]
Rybelsus, containing semaglutide	T2D	0.6 [0.3–0.8]	0.5 [0.3–0.7]	0.3 [0.1–0.4]	0.3 [0.2–0.5]	0.1 [0.0–0.1]

*CI* Confidence interval, *GLP-1* Glucagon-like peptide-1, *GIP *Glucose-dependent insulinotropic polypeptide, *WL *Weight loss, *CRR *Cardiovascular risk reduction, *T2D *Type 2 diabetes

^a^Authorised indications for use in the UK [12]

Data shown are weighted estimates of the proportion (with 95% CI) of adults in Great Britain reporting past-year use of different GLP-1 and GLP-1/GIP medications, overall (i.e. for any reason) and stratified by the reason for use. Note that reasons are not mutually exclusive, except ‘exclusively to support weight loss’, which excludes participants reporting use for any other reason.

Estimates stratified by gender are provided in Additional file 2: Table S3

There were some subgroup differences in the use of GLP-1 or GLP-1/GIP medication to support weight loss (Table 2). Prevalence was more than twice as high among women than men (4.0% vs. 1.7%; OR = 2.40 [1.62–3.56])—with an even greater difference in the proportion using them exclusively for weight loss (2.8% [70.0% of female users] vs. 0.6% [35.3% of male users]; OR = 4.44 [2.43–8.15]). There was a non-linear (inverted U-shaped) association with age, with the highest prevalence of use of GLP-1 or GLP-1/GIP medication to support weight loss among those in mid-life (e.g. 4.2% among those aged 45 and 55) and lower prevalence in early adulthood (e.g. 1.2% among those aged 18) and later life (e.g. 1.5% among those aged 75). Prevalence was also higher among those who reported moderate/severe psychological distress (3.7% vs. 2.4% among those reporting no/low distress; OR = 1.62 [1.13–2.32]). It also appeared to be higher among those who reported a history of eating disorders (4.4% vs. 2.1% among those who did not; OR = 2.12 [0.69–6.53]), but there was substantial imprecision in the estimate for this comparison given information on eating disorders was only collected in one of the three survey waves and only a small proportion (4.2%) reported having received a diagnosis (Additional file 2: Table S1). There were no notable overall differences by ethnicity, socioeconomic markers, smoking status, or level of alcohol consumption. However, among men, prevalence appeared to be higher among those from less advantaged socioeconomic groups (i.e. those from occupational social grades C2DE, those finding it difficult to manage financially, and those not in work due to long-term illness or disability), whereas prevalence was more similar or showed the opposite pattern among women (Additional file 2: Table S2).

**Table 2:** Use of GLP-1 receptor agonists and GLP-1/GIP compounds to support weight loss, and interest in using weight-loss medication, by participant characteristics

	**Used GLP-1 or GLP-1/GIP medication to support weight loss in the past year**^**a**^		**Interested in using weight-loss medication in the next year**^**b**^	
	**% [95% CI]**	**OR [95% CI]**^**c**^	**% [95% CI]**	**OR [95% CI]**^**c**^
Gender				
Man	1.7 [1.1–2.3]	Ref	5.1 [4.2–6.1]	Ref
Woman	4.0 [3.2–4.8]	2.40 [1.62–3.56]	8.9 [7.7–10.1]	1.85 [1.44–2.38]
Age (years)^d^				
18	1.2 [0.7–2.1]	Ref	5.5 [3.8–7.9]	Ref
25	1.8 [1.2–2.7]	1.53 [1.31–1.74]	6.8 [5.3–8.6]	1.26 [1.11–1.40]
35	3.1 [2.4–3.9]	2.64 [2.03–3.26]	8.7 [7.5–10.0]	1.67 [1.30–2.04]
45	4.2 [3.4–5.3]	3.74 [2.64–4.85]	9.7 [8.4–11.3]	1.91 [1.35–2.46]
55	4.2 [3.4–5.2]	3.71 [2.42–4.99]	8.6 [7.3–10.1]	1.66 [1.07–2.25]
65	2.9 [2.2–3.7]	2.46 [1.46–3.46]	5.8 [4.8–6.9]	1.07 [0.58–1.56]
75	1.5 [1.0–2.4]	1.27 [0.51–2.03]	3.2 [2.4–4.4]	0.57 [0.12–1.02]
Ethnicity				
White	2.9 [2.4–3.4]	Ref	6.6 [5.8–7.4]	Ref
Minority ethnic group	2.9 [1.5–4.2]	1.05 [0.64–1.73]	9.6 [7.0–12.3]	1.30 [0.91–1.86]
Occupational social grade				
ABC1 (more advantaged)	3.1 [2.5–3.7]	Ref	6.8 [5.9–7.8]	Ref
C2DE (less advantaged)	2.6 [1.7–3.4]	0.85 [0.57–1.27]	7.4 [6.0–8.7]	1.09 [0.84–1.41]
Financial situation				
Living comfortably	2.9 [2.1–3.7]	Ref	4.5 [3.4–5.7]	Ref
Doing alright	2.6 [1.8–3.4]	0.88 [0.57–1.34]	6.4 [5.1–7.7]	1.26 [0.89–1.78]
Just about getting by	2.9 [1.8–4.1]	0.99 [0.61–1.62]	8.7 [7.0–10.5]	1.98 [1.40–2.80]
Finding it quite difficult	3.3 [1.5–5.1]	1.11 [0.58–2.12]	9.6 [6.6–12.6]	2.02 [1.28–3.19]
Finding it very difficult	3.7 [1.2–6.3]	1.17 [0.55–2.50]	11.7 [7.6–15.7]	2.01 [1.16–3.48]
Economically inactive due to long-term illness or disability				
No	2.8 [2.3–3.3]	Ref	6.7 [5.9–7.5]	Ref
Yes	4.3 [1.7–6.9]	1.26 [0.66–2.43]	13.4 [9.1–17.7]	1.85 [1.19–2.88]
Smoking status				
Never	2.6 [2.0–3.3]	Ref	6.4 [5.4–7.4]	Ref
Former	3.6 [2.6–4.5]	1.35 [0.92–1.97]	8.1 [6.5–9.6]	1.26 [0.93–1.71]
Current	2.4 [1.2–3.7]	0.87 [0.48–1.56]	7.5 [5.3–9.6]	1.09 [0.76–1.56]
Level of alcohol consumption (AUDIT-C)^e^				
0	3.2 [2.4–4.3]	Ref	8.3 [7.0–9.9]	Ref
3	2.5 [2.0–3.3]	0.79 [0.41–1.17]	6.0 [5.0–7.2]	0.76 [0.49–1.02]
5	2.5 [1.9–3.3]	0.82 [0.39–1.26]	5.9 [4.9–7.1]	0.77 [0.46–1.07]
8	3.0 [2.2–4.1]	1.12 [0.66–1.58]	7.4 [6.0–9.0]	0.99 [0.64–1.33]
11	3.9 [2.0–7.4]	1.67 [0.92–2.42]	10.0 [6.6–14.8]	1.40 [0.81–1.99]
Past-month psychological distress				
None/low	2.4 [1.8–2.9]	Ref	5.2 [4.3–6.1]	Ref
Moderate/severe	3.7 [2.8–4.6]	1.62 [1.13–2.32]	10.0 [8.5–11.4]	1.81 [1.40–2.35]
History of eating disorders^f^				
No	2.1 [1.4–2.9]	Ref	7.3 [5.8–8.7]	Ref
Yes	4.4 [0.1–8.8]	2.12 [0.69–6.53]	13.2 [4.0–22.4]	1.59 [0.66–3.82]

*CI* Confidence interval, *OR* Odds ratio, *GLP-1 *Glucagon-like peptide-1, *GIP *Glucose-dependent insulinotropic polypeptide

^a^Among adults in Great Britain (*n* = 5893)

^b^Among those who had not used a GLP-1, GLP-1/GIP, or other medication for weight loss in the past year (*n* = 5657)

^c^Adjusted for age and gender (analyses by age are adjusted for gender only and analyses by gender are adjusted for age only)

^d^Predicted estimates from logistic regression models with age modelled using restricted cubic splines. Note that the models used to derive these estimates included data from participants of all ages, not only those who were aged exactly 18, 25, 35, 45, 55, 65, or 75 years

^e^Predicted estimates from logistic regression models with AUDIT-C score modelled using restricted cubic splines. Note that the models used to derive these estimates included data from all participants who provided data on AUDIT-C, not only those who scored exactly 0, 3, 5, 8, or 11

^f^History of eating disorders was only assessed in February 2025; analyses were restricted to participants surveyed in this wave

Estimates stratified by gender are provided in Additional file 2: Table S2 (use in the past year) and Additional file 2: Table S4 (interest). Goodness of fit statistics are provided in Additional file 2: Table S5

### Types of GLP-1 and GLP-1/GIP medications being used

The most commonly used medication was Mounjaro (tirzepatide; 2.3%)—a GLP-1/GIP medication, followed by GLP-1 receptor agonists Ozempic (semaglutide; 1.3%), Wegovy (semaglutide; 0.8%), Rybelsus (semaglutide; 0.6%), and Saxenda (liraglutide; 0.4%; Table 1). Of note, Mounjaro was most commonly used to support weight loss; it was three times more prevalent for this reason than the next most popular medication (2.0% vs. Wegovy at 0.6%; Table 1).

Among those who used a GLP-1 or GLP-1/GIP medication for weight loss in the past year, 85.0% [79.3–90.7%] used a medication licensed for weight loss in Great Britain—69.5% [62.0–67.0%] reported using Mounjaro, 20.0% [12.9–27.0%] Wegovy, and 3.3% [0.0–6.8%] Saxenda (note that participants could select multiple medications, so these values sum to more than the total; 19.4% [12.7–26.1%] of those who reported using GLP-1 or GLP-1/GIP medication for any reason reported using more than one GLP-1 or GLP-1/GIP medication—0.7% [0.5–1.0%] of all participants). Among those who used a GLP-1 or GLP-1/GIP medication exclusively for weight loss, those numbers were 91.4% [85.6–97.2%], 80.2% [71.9–88.6%], 21.4% [12.4–30.3%], and 0.9% [0.0–2.6%], respectively.

### Interest in using weight-loss medications

**Fig. 1:**
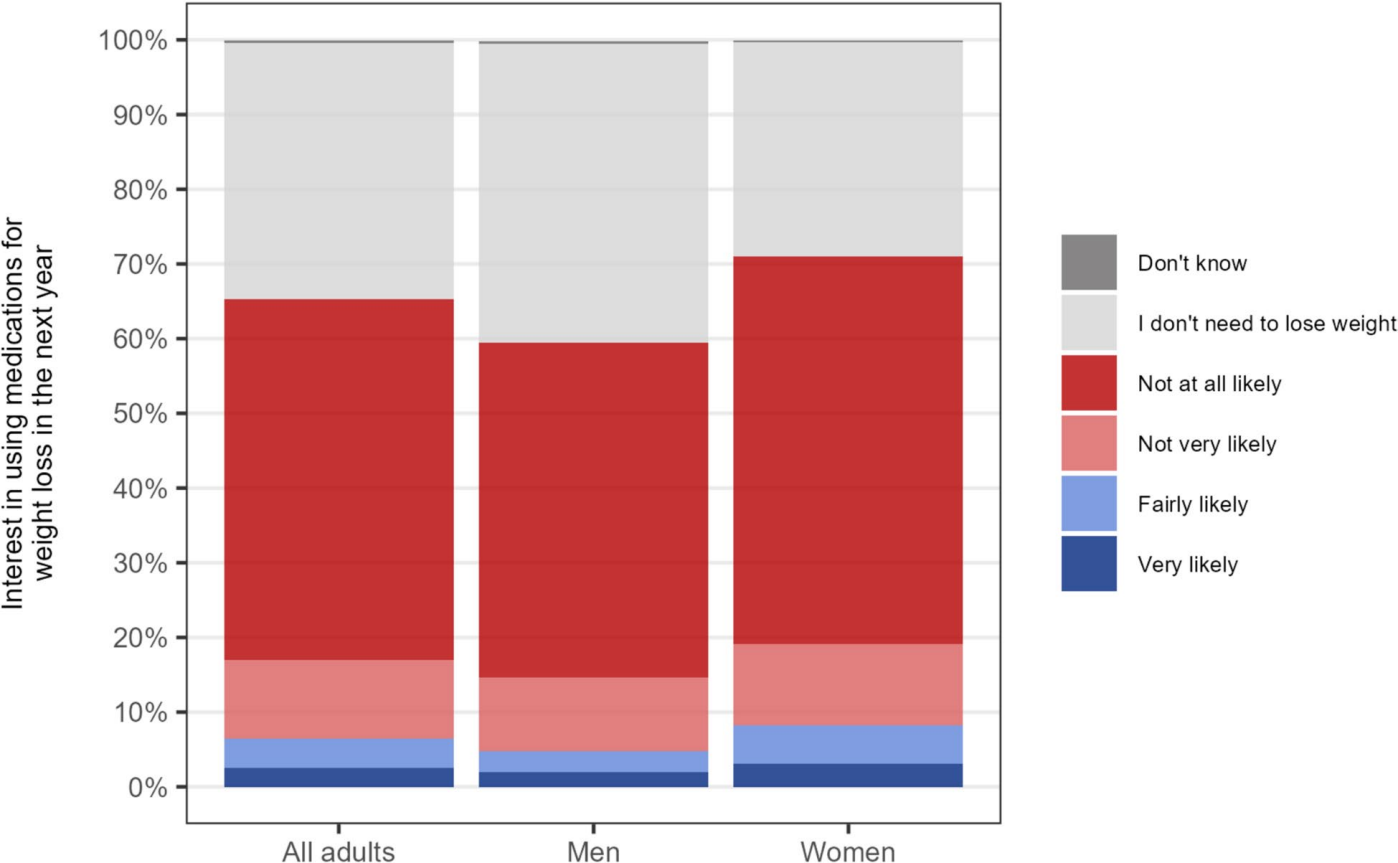
Interest in using weight-loss medication among adults (≥ 18 years) in Great Britain who have not done so in the past year, overall and by gender

Among participants who had not used a GLP-1, GLP-1/GIP, or other medication for weight loss in the past year (96.0% [95.5–96.6%] of the sample), 6.5% [5.7–7.3%] said they would be likely to consider using a weight-loss medication in the next year (2.5% [2.1–3.0%] very likely and 4.0% [3.4–4.6%] fairly likely; Fig. 1). This is approximately 3.3 million adults in Great Britain (53.7 million adults × 96.0% not used medication to support weight loss in the past year × 6.5%), of whom around 1.3 million (53.7 million × 96.0% × 2.5%) are very likely to consider it.

Interest in using weight-loss medications differed according to participant characteristics (Table 2). Some of these differences mirrored patterns observed for past-year use of GLP-1 and GLP-1/GIP medications for weight loss. The proportion who said they would be likely to consider using weight-loss medications in the next year was higher among women than men (8.9% vs. 5.1%; OR = 1.85 [1.44–2.38]; Fig. 1), those in mid-life (e.g. 9.7% among those aged 45 vs. 5.5% and 3.2% among those aged 18 and 75, respectively), and those who reported moderate/severe psychological distress (10.0% vs. 5.2% among those reporting no/low distress; OR = 1.81 [1.40–2.35]). It also appeared to be higher among those who reported a history of eating disorders (13.2% vs. 7.3% among those who did not; OR = 1.59 [0.66–3.82]), but again, there was substantial imprecision.

There were also some subgroup differences that were not observed for past-year use (Table 2). Interest in using weight-loss medications in the next year was higher among those in less favourable financial situations (e.g. 11.7% among those who reported finding it very difficult to manage financially vs. 4.5% among those living comfortably; OR = 2.01 [1.16–3.48]) and among those who were not in work due to long-term illness or disability (13.4% vs. 6.7% of those not in this situation; OR = 1.85 [1.19–2.88]). It also appeared to be slightly higher among those from minority ethnic groups (9.6% vs. 6.6% among white participants; OR = 1.30 [0.91–1.86]), but this difference was uncertain. There were no notable differences by occupational social grade, smoking status, or level of alcohol consumption.

## Discussion

Our data suggest that in the first quarter of 2025, an estimated 4.9 million adults in Great Britain—nearly one in ten—either had recently used medication to support weight loss or were interested in doing so in the near future. Of these, approximately 1.6 million adults used GLP-1 or GLP-1/GIP medications for weight management, with 910,000 (~ 60%) using them exclusively for this purpose. The majority reported using GLP-1 or GLP-1/GIP medications that are licensed for weight loss in Great Britain, most commonly Mounjaro (tirzepatide). Use and interest were more prevalent among women, people in mid-life, and those reporting past-month psychological distress, and also appeared higher among those with a history of eating disorders. Interest was also higher among people facing greater socioeconomic disadvantage, including those in financial difficulty or unable to work due to long-term illness or disability.

These data provide important insights into the emerging landscape of GLP-1 and GLP-1/GIP medication use and potential future demand in Great Britain. The substantial level of current use, combined with even greater levels of interest, highlights growing public awareness of pharmacological options for weight management. The gap between interest and current use suggests there may be unmet demand. However, in the absence of data on people’s underlying medical risk factors, it is not possible to understand the extent to which this reflects ‘true’ medical need, e.g. meeting either current NICE criteria for weight loss medication or a lower threshold at which health benefits outweigh the harms. While the numbers we estimate are for Great Britain as a whole, it is noteworthy that the number using these medications for weight loss far exceeds the initial expectation for NHS England to provide treatment for 220,000 people in the first 3 years, which is likely to have important consequences for NHS budgets, although our estimate is less than the number eligible according to NICE guidelines (3.4 million) [14]. Part of the discrepancy may reflect prescribing outside the NHS. Recent data from life-sciences analytics company IQVIA suggest that approximately 1.4 million people in the UK now access GLP-1 medications privately each month, predominantly through online pharmacies [40]. This estimate is relatively close to our estimate of 1.6 million using for weight loss and supports the notion that most GLP-1 for weight loss in the UK is from private, rather than NHS provision. It is unclear to what extent private use is driven by health concerns—and falls within either NICE recommendations or licensed use—or desire to lose weight for other reasons. Nonetheless, this trend is consistent with broader societal shifts towards medicalised approaches to managing obesity [11, 41] and points to a need for healthcare systems and policymakers to decide how best to manage a continued surge in demand.

Consistent with previous studies showing greater uptake of weight-loss treatments among women [24], we observed higher use and interest among women than men. This may reflect a combination of factors, including greater social pressures on women regarding body image and weight [25], as well as gender differences in healthcare-seeking behaviour [42]. Women have much higher prevalence of severe obesity than men [2], and are typically more likely to engage with health (and preventive) services [43], engage with weight management services, and discuss weight loss with healthcare professionals [24], which may facilitate greater access to emerging treatments like GLP-1 and GLP-1/GIP medications.

We also identified socioeconomic patterns. Although current use of GLP-1 and GLP-1/GIP medications for weight loss was relatively consistent across socioeconomic groups, this did vary by gender. Among men, use was more common in less advantaged groups, while among women, use was relatively consistent across socioeconomic strata. This may reflect higher prevalence of obesity-related metabolic comorbidities among men compared to women, which may also be socially patterned [44, 45]. For men, from a health inequalities perspective, it is encouraging that uptake is higher among people from less advantaged groups. It is also unusual for the adoption of an expensive new medication or new medical technology, and this finding warrants further scrutiny and exploration. For women, one possible explanation is that women may be more likely than men to access GLP-1 and GLP-1/GIP medications through private routes, which often require substantial out-of-pocket costs [19] and may be less accessible to those with fewer financial resources. Further research is needed to explore these findings more fully.

Interest in future use was higher among those experiencing financial hardship or unemployment due to long-term illness or disability. The greater interest among groups disproportionately affected by severe obesity [2, 46] and its related comorbidities may reflect a recognition of the potential health benefits of weight-loss medications for these people [27, 28]. It may also reflect a lack of perceived viable alternatives to lose weight: lifestyle interventions for weight management (even very intensive, well-designed, multicomponent ones) have a very modest impact on weight loss [47, 48].

Use of GLP-1 and GLP-1/GIP medications for weight loss and interest in future use were also higher among those experiencing psychological distress and those reporting a history of eating disorders. This may reflect the well-documented bidirectional relationship between mental health conditions—such as depression, anxiety, and binge eating disorder—and obesity [49, 50]. Mental health disorders are also more prevalent among women [51], which may contribute to observed gender differences in GLP-1 and GLP-1/GIP medication use and interest. Individuals with higher levels of psychological distress may be more vulnerable to weight-related stigma or internalised weight bias [52], leading to greater concern about appearance and heightened motivation to seek pharmacological interventions. Greater body shame, weight concerns, anti-fat bias, and disordered eating behaviours have all been linked to greater interest in using GLP-1 medications [53]. However, the direction of these associations is unclear and may be bidirectional. Emerging evidence has raised concerns about potential adverse mental health effects associated with GLP-1 medication use, including increased psychological distress or suicidal ideation in some users, although data are currently limited and inconclusive [54, 55]. These patterns highlight the importance of further research to better understand the relationship between mental health and both actual and intended use of GLP-1 medications for weight management.

Another notable finding is the substantial proportion of past-year users who reported using GLP-1 medications that are not licensed for weight loss (15.0% of all those who reported using a GLP-1 or GLP-1/GIP medication to support weight loss; 8.6% of those reporting use exclusively for weight loss), indicating off-label prescribing [56] or procurement through non-medical channels [57, 58]. This may highlight potential issues in prescribing practices, patient understanding, and/or regulatory oversight. Some off-label prescribing should be expected: in the UK, liraglutide has an indication for weight loss only, but NICE guidelines list it as a treatment option for type 2 diabetes [59]. While off-licence prescribing can be common in some areas of medicine [60], it can also pose safety risks [61]—particularly when medications are accessed without appropriate clinical supervision. There are concerns about GLP-1 and GLP-1/GIP medications being purchased without a prescription through unregulated or illicit channels, with very light medical supervision or being used outside their licensed use (i.e. at lower BMI). Further work is needed, but this data raises further questions about their actual use and whether appropriate safety standards are in place and being adhered to. Depending on how these medications are being accessed, public health messaging warning of the potential harms of purchasing online or from other non-medical outlets may also be helpful.

The rising demand for GLP-1 and GLP-1/GIP medications to support weight loss presents significant challenges for healthcare systems such as the NHS. Responding to this demand safely and equitably, while focusing on those with medical need, will require difficult decisions about access, prioritisation, and resource allocation. These discussions are already underway and have led to a new proposition about how to identify those with clinical need who would benefit most from these drugs, although this is not without controversy [62, 63]. Although widespread adoption of GLP-1 and GLP-1/GIP medications has the potential to improve population health by reducing obesity-related diseases, the financial implications are considerable. These medications are expensive [11], are likely to require long-term use, and scaling up provision could place a heavy burden on NHS budgets already strained by the management of chronic conditions and recent structural reorganisations [64]. Equally the majority of the population thought they were not interested in using the medications, and while attitudes may change, as with other medical interventions this might suggest limits on public willingness to use them. While GLP-1 and GLP-1/GIP medications are generally considered cost-effective for treating obesity in targeted populations [65–67], alternative strategies—such as expanding bariatric surgery access, enhancing lifestyle intervention programmes, pharmaceutical management of cardiometabolic comorbidities, or population-level policies—should also be considered. These may not yet have achieved an equivalent scale of impact compared to GLP-1s, but some have not been tried at scale or with the intensity that is required, but may offer more cost-effective, long lasting or equitable solutions [11]. Policymakers will need to balance the benefits of increasing GLP-1 and GLP-1/GIP medication access against broader public health investments, which have been slow to be implemented (e.g. advertising and product promotion restrictions [64] that are not yet in place, and which have been further delayed). Furthermore, it will be essential to ensure that access is equitable, clinically justified, and aligned with evidence-based guidelines to prevent widening health inequalities or encouraging inappropriate prescribing. In addition, there is a need to ensure these medications remain available to those who need them for reasons other than weight loss (over a third of users in 2024, according to our data; ~ 1.5 million people).

A key strength of this study is the relatively large, nationally representative sample. In addition, the inclusion of demographic, socioeconomic, and psychological factors provides a detailed picture of patterns of use and potential demand within different population subgroups. There were also several limitations. All data were self-reported and relied on recall of the past year, introducing scope for bias. Given the cross-sectional nature of the study, we were unable to disentangle directional associations with time-varying sample characteristics and may have been unable to detect some such associations. For example, while we did not observe an association between alcohol consumption and past-year use of GLP-1 and GLP-1/GIP medications, it is possible that those who used these medications initially had higher consumption but experienced reductions in alcohol consumption between initiating medication use and completing the survey [8]. Due to limited availability of funding for the GLP-1 survey items, we were unable to collect more detailed information about how and why people were accessing these medications. No data were available on height and weight, so we were unable to explore differences by BMI status or the extent to which observed subgroup differences were explained by differences in BMI and cannot make assessments about the appropriateness of use against medical criteria for use. This is an important area for future studies to explore. We also did not ask specifically about all GLP-1 medications licensed in the UK, which may have resulted in incomplete information on the types of medications used and caused us to underestimate the overall prevalence of use.

Further research is needed to explore the source of GLP-1 and GLP-1/GIP medications (e.g. NHS, private prescription in person, post/internet with a prescription, post/internet with no formal prescription) and to assess the appropriateness of use (i.e. the extent to which people who are using these drugs for weight loss fall within the indications of the NICE guidelines). Regularly including these types of questions in a representative, repeat cross-sectional household survey like the Smoking and Alcohol Toolkit Study, alongside assessment of height and weight, would offer insights into how population-level use of, and demand for, GLP-1 and GLP-1/GIP medications for weight loss is evolving over time and, importantly, how it relates to people’s BMI. Qualitative research is also important for a richer understanding of people’s experiences of accessing and using these medications.

## Conclusions

This study highlights substantial demand for GLP-1 and GLP-1/GIP medications to support weight loss in Great Britain. As drugs become more available, equitable access to these treatments should be prioritised to meet the needs of all population groups, particularly those who may face barriers to access despite high interest. At the same time, healthcare systems must be prepared for the potential pressures on capacity and budgets. To support safe, effective, and equitable use, there is a clear need for regular, population-level monitoring—not only of the prevalence and patterns of use, but also of access, appropriateness, health outcomes, and broader system impacts. Such surveillance will be essential to inform responsive healthcare planning, guide and evaluate policy decisions, and ensure that these treatments deliver sustainable benefits without widening health inequalities or overburdening healthcare resources.

## Supplementary Information

Additional file 1: Survey measures.

Additional file 2: Tables S1–S5. Table S1 Sample characteristics. Table S2 Use of GLP1s for weight loss, gender-stratified. Table S3 Types of GLP1s used, gender-stratified. Table S4 Interest in using weight loss medication, gender-stratified. Table S5 Goodness of fit statistics.

## Data Availability

Data used in these analyses are available on Open Science Framework ([https://osf.io/r2whq/] (https:/osf.io/r2whq)), with age provided in bands to preserve participant anonymity.

## Abbreviations

ABC1: More advantaged social grades (includes managerial, professional, and upper supervisory occupations)
AUDIT-C: Alcohol Use Disorders Identification Test—consumption
BMI: Body mass index
C2DE: Less advantaged social grades (includes manual routine, semi-routine, lower supervisory, state pension, and long-term unemployed)
GIP: Glucose-dependent insulinotropic polypeptide
GLP-1: Glucagon-like peptide-1
GP: General practitioner
K6: Kessler Psychological Distress Scale
NHS: National Health Service
NICE: National Institute for Health and Care Excellence
