# Supplementary material for: Prevalence of use and interest in using glucagon-like peptide-1 receptor agonists for weight loss: a population study in Great Britain
Source: BMC Med. 2026 Jan 8;24:1. doi: 10.1186/s12916-025-04528-7 (PMC12781702; doi:10.1186/s12916-025-04528-7)
Supplement: Supplementary file 1 — Additional file 1: Survey measures. [file 12916_2025_4528_MOESM1_ESM.pdf]

Additional file 1

Past-year use of GLP-1 receptor agonists and GLP-1/GIP compounds

FILTER: ASK ALL

QAGREE. The next few questions are about personal health related matters, such as taking medication. A "Prefer not say" option is available for you to select at your discretion.

Participation is voluntary and you may withdraw your consent at any time. Your responses are used for research purposes only. Answers are confidential and will only be reported as aggregated statistics, when combined with responses from others taking part. Your personal data will be held for no longer than 12 months.

Do you agree to continue with these questions?

SINGLE CODE

1. Yes - I agree
2. No - I do not agree – **SKIP TO PAST QGLP1 AND QGLP2**

FILTER: ASK ALL

QGLP1. In the last 12 months have you taken any medication to help you with any of the following, or not?

MULTI CODE 1-3, RANDOMISE CODES 1-3

1. Medication for Type 2 Diabetes, excluding insulin, to help manage your blood sugar levels
2. Medication to lower the risk of heart disease
3. Medication to support weight loss / reduce food cravings
4. I have not taken medication for any of these reasons **(SINGLE CODE)**
5. Can't remember **(DO NOT READ OUT)**

FILTER: ASK IF QGLP1=1-3

QGLP2. In the last 12 months, which, if any, of the following types of medication have you taken for [TEXT SUBS IF GLP'1':Type 2 Diabetes, TEXT SUBS IF GLP'2': to lower the risk of heart disease, TEXT SUBS IF GLP'3': for weight loss] ?

MULTI CODE 1-5, RANDOMISE CODES 1-4

1. Saxenda, containing Liraglutide
2. Ozempic, containing Semaglutide
3. Wegovy, containing Semaglutide
4. Mounjaro, containing Tirzepatide
5. Rybelsus, containing Semaglutide
6. Another type of medication (please specify)
7. Don't know (DO NOT READ OUT)

Interest in using medication for weight loss

FILTER: QGLP1= NOT 3

QWL_218. In the next 12 months, how likely, if at all, are you to consider using a weight loss medication to help you lose weight?

SINGLE CODE

1. Very likely
2. Fairly likely
3. Not very likely
4. Not at all likely
5. I don't need to lose weight
6. Don't know **(DO NOT READ OUT)**

Additional file 1

<u>Sociodemographic characteristics</u>

sexz. Which of the following best describes how you think of yourself?
1. Male
2. Female
3. In another way

actage. Enter exact age
1. <OPEN ENDED>
2. Refused

FILTER: actage=2
agez. Which age group applies to you?
1. Under 16
2. 16-17
3. 18-24
4. 25-34
5. 45-54
6. 55-59
7. 60-64
8. 65-74
9. 75+

ethnicity. Which group on this card do you consider you belong to? Please read out the letter.

1. A. White - English / Welsh / Scottish / Northern Irish / British
2. B. White - Irish
3. C. White - Gypsy or Irish Traveller
4. D. White - Any other White background
5. E. Mixed - White and Black Caribbean
6. F. Mixed - White and Black African
7. G. Mixed - White and Asian
8. H. Mixed - Any other Mixed / multiple ethnic background
9. I. Asian/Asian British - Indian
10. J. Asian/Asian British - Pakistani
11. K. Asian/Asian British - Bangladeshi
12. L. Asian/Asian British - Chinese
13. M. Asian/Asian British - Any other Asian background
14. N. Black - African
15. O. Black - Caribbean
16. P. Black - Any other Black / African / Caribbean background
17. Q. Arab
18. R. Any other ethnic group
19. Don't know
20. Refused

ARQ1. How well would you say you yourself are managing financially these days? Would you say you are...

SINGLE CODE FORWARD/REVERSE 1-5

1. Living comfortably
2. Doing alright
3. Just about getting by
4. Finding it quite difficult
5. Finding it very difficult

work. Which of these applies to you?

1. Have paid job – Full time (30+ hours per week)
2. Have paid job – Part time (8-29 hours per week)
3. Have paid job – Part time (Under 8 hours per week)
4. Not working – Housewife
5. Self-employed
6. Full time student
7. Still at school
8. Unemployed and seeking work
9. Retired
10. Not in paid work for other reason
11. Not in paid work because of long term illness or disability
12. Refused

Social grade assigned by interviewer after determining:

- Details of the chief income earner in the household's occupation (present or last)
- Chief income earner in the household's job title (present or last)
- Chief income earner in the household's qualifications / apprenticeships
- The number the chief income earner in the household is responsible for

Health-related behaviours

Q632A1. Which of the following best applies to you?

Please note we are referring to cigarettes and other kinds of tobacco that you set light to and NOT electronic or 'heat-not-burn' cigarettes.

SINGLE CODE

1. I smoke cigarettes (including hand-rolled) every day
2. I smoke cigarettes (including hand-rolled), but not every day
3. I do not smoke cigarettes at all, but I do smoke tobacco of some kind (eg. Pipe, cigar or shisha)
4. I have stopped smoking completely in the last year
5. I stopped smoking completely more than a year ago

6. I have never been a smoker (i.e. smoked for a year or more)
7. Don't know **(DO NOT READ OUT)**

These next few questions ask about the alcohol you have drunk **in the last 6 months**, including about how many standard drinks you have consumed. Please note that 1 standard drink equals 1 unit of alcohol. So, for example, a small glass of wine or a single measure of spirits is 1 standard drink, while a pint of regular beer or lager is equal to 2 standard drinks or 2 units, and a bottle of wine is equal to 9 units If you are unsure, please ask me to help you work it out.

audit1. How often do you have a drink containing alcohol?
READ OUT IF NECESSARY

0. Never
1. Monthly or less
2. 2 to 4 times a month
3. 2 to 3 times a week
4. 4 to 5 times a week
5. 6 or more times a week
6. Don't know (DO NOT READ OUT)
7. Refused (DO NOT READ OUT)

ASK ALL EXCEPT 0 AT audit1
audit2. How many standard drinks containing alcohol do you have on a typical day when you are drinking?
READ OUT IF NECESSARY
INTERVIEWER: IF RESPONDENT SAYS 'DON'T KNOW' ENCOURAGE THEM TO GIVE BEST ESTIMATE

0. 1 to 2
1. 3 to 4
2. 5 to 6
3. 7 to 9
4. 10 to 12
5. 13 to 15
6. 16 or more
7. Don't know (DO NOT READ OUT)
8. Refused (DO NOT READ OUT)

ASK ALL EXCEPT 0 AT audit1
audit3. How often do you have six or more standard drinks on one occasion?
READ OUT IF NECESSARY

0. Never
1 Less than monthly
2 Monthly
3 Weekly
4 Daily or almost daily
5. Don't know (DO NOT READ OUT)
6. Refused (DO NOT READ OUT)

Additional file 1

Mental health

SY03.
During the past 30 days, about how often, if at all, did you feel…?
SINGLE CODE PER STATEMENT

STATEMENTS [RANDOMISE]
1. …nervous?
2. …hopeless?
3. …restless or fidgety?
4. …so depressed that nothing could cheer you up?
5. …that everything was an effort?
6. …worthless?

ANSWER CODES [FORWARD AND REVERSE]
1. All of the time
2. Most of the time
3. Some of the time
4. A little of the time
5. None of the time
6. Don't know **[FIX]**
7. Prefer not to say **[FIX]**

SY01. Now please look at the health conditions listed on this screen.
Since the age of 16, which of the following, if any, has a doctor or health professional ever told you that you had? Please select all that apply.
As a reminder any information you give is strictly confidential and will be used for research purposes only.
MULTICODE, RANDOMISE
1. Depression
2. Anxiety
3. Obsessive Compulsive Disorder
4. Panic Disorder or a phobia
5. Post-traumatic Stress Disorder
6. Psychosis
7. Personality Disorder
8. Attention Deficit Hyperactivity Disorder
9. An Eating Disorder
10. Alcohol Misuse or Dependence
11. Drug Use or Dependence
12. Problem Gambling
13. None of these **[FIX, SINGLE CODE]**
14. Don't know **[FIX, SINGLE CODE]**
15. Prefer not to say **[FIX, SINGLE CODE]**
